# Experimental and Numerical Study of Healing Effect on Delamination Defect in Infusible Thermoplastic Composite Laminates

**DOI:** 10.3390/ma16206764

**Published:** 2023-10-19

**Authors:** Paulius Griskevicius, Kestutis Spakauskas, Swarup Mahato, Valdas Grigaliunas, Renaldas Raisutis, Darius Eidukynas, Dariusz M. Perkowski, Andrius Vilkauskas

**Affiliations:** 1 Department of Mechanical Engineering, Kaunas University of Technology, Studentu St. 56, LT-51424 Kaunas, Lithuania; kestutis.spakauskas@ktu.lt (K.S.); swarup.mahato@ktu.lt (S.M.); 2 Institute of Mechatronics, Kaunas University of Technology, Studentu St. 56, LT-51424 Kaunas, Lithuania; valdas.grigaliunas@ktu.lt (V.G.); darius.eidukynas@ktu.lt (D.E.); andrius.vilkauskas@ktu.lt (A.V.); 3 Ultrasound Research Institute, Kaunas University of Technology, K. Barsausko St. 59, LT-51423 Kaunas, Lithuania; renaldas.raisutis@ktu.lt; 4 Faculty of Mechanical Engineering, Bialystok University of Technology, 45C Wiejska St., 15-351 Białystok, Poland; d.perkowski@pb.edu.pl

**Keywords:** fiber reinforced thermoplastic composite, delamination, thermal healing, compression-after-impact, cohesive zone modelling

## Abstract

The integrity of delaminated composite structures can be restored by introducing a thermally-based healing effect on continuous fiber-reinforced thermoplastic composites (CFRTPC). The phenomenon of thermoplastics retaining their properties after melting and consolidation has been applied by heating the delaminated composite plates above their glass transition temperature under pressure. In the current investigation, the composite is comprised of Methyl methacrylate (MMA)-based infusible lamination resin combined with benzoyl peroxide initiator, which polymerizes into a Polymethyl methacrylate (PMMA) matrix. For the reinforcement, unidirectional 220 gr/m^2^ glass filament fabric was used. Delamination damage is artificially induced during the fabrication of laminate plates. The distributed delamination region before and after thermally activated healing was determined by using non-destructive testing with active thermography. An experimental approach is employed to characterize the thermal healing effect on mechanical properties. Experimentally determined technological parameters for thermal healing have been successfully applied to repair delamination defects on composite plates. Based on the compression-after-impact (CAI) test methodology, the intact, damaged, and healed composite laminates were loaded cyclically to evaluate the healing effect on stiffness and strength. During the CAI test, the 3D digital image correlation (DIC) technique was used to measure the displacement and deformation fields. Experimental results reveal the difference between the behavior of healed and damaged specimens. Additionally, the numerical models of intact, damaged, and healed composite laminates were developed using the finite element code LS-Dyna. Numerical models with calibrated material properties and tie-break contact constants provide good correlation with experimental results and allow for the prediction of the mechanical behavior of intact, damaged, and healed laminated plates. The comparison analysis based on CAI test results and modal characteristics obtained by the 3D Laser Doppler Vibrometer (Polytec GmbH, Karlsbad, Germany) proved that thermal healing partially restores the mechanical properties of damaged laminate plates. In contrast, active thermography does not necessarily indicate a healing effect.

## 1. Introduction

Continuous fiber-reinforced thermoplastic composites (CFRTPC) are preferred in various industries because of their recyclability properties [1,2], possibility of thermal welding [3,4], easy repair [5,6,7], high fracture toughness [8], damage tolerance [9], and applicability in additive manufacturing technology with in-situ consolidation of thermoplastic automated fiber placement processes [10]. The recent development of thermoplastic liquid resin systems has increased the trend toward using thermoplastic composites [7,11,12].

The matrix being brittle makes the composite laminate sensitive to impact. Low-velocity impact loads initiate matrix cracking and delamination [13]. Both of the mentioned damages are hardly detected by typical visual inspections, and in the case of fatigue load, barely visible internal damages (microcracks or delamination) can result in the macro failure of the entire composite component. Monitoring and assessing structural integrity is essential for economic and safety reasons. Early detection of this phenomenon using non-destructive testing (NDT) techniques [14] enables predicting the residual strength of the composite structure and adhesive joints [15,16,17] and selecting appropriate methods for repair [5,6,7,18,19]. Identification and mitigation of damage-tailored repair of composite components is a time-consuming and costly procedure. A significant advantage of thermoplastic composites is their capability to facilitate repairs of delamination or matrix cracking damage without removing the affected section [12,20,21]. As a result of these problems, there is an increasing demand for polymer composites with self-healing or damage-recovery functionality [12,22,23,24,25].

Most fully autonomous self-healing systems are typically based on healing agents (microcapsules [26,27] or vascular systems [28,29]) inserted into the matrix before the consolidation. Among all other healing agents, PMMA gains more popularity for its high chemical stability, better compatibility with most of the host material, and good mechanical properties [30,31]. Ahangaran et al. [27] used 10 wt% PMMA healing capsules for the epoxy composites. They achieved 80% of the healing efficiency of fracture toughness in a tapered double cantilever beam specimen. The primary limitation of these fully autonomous healing systems lies in their single-use options. Damage recovery performed by external triggering (heat, pressure, light, or chemical substances) refers to non-autonomous but multiple healing events. Post et al. [22,32] state that they were able to heal delamination and achieve the strength of the healed specimen with an ionomer interlayer close to the strength of the pristine specimen. While thermal self-healing of composites holds great promise, for their successful implementation, there are several challenges and limitations, such as slow healing rates, limited healing efficiency, and difficulty in achieving repeatable healing cycles [33].

The aim of the work was to combine experimental and numerical approaches to assess the effectiveness of damage recovery on delamination defects in infusible thermoplastic composite laminates through mechanical compression after impact (CAI) and non-destructive testing. In this work, a comparative study is performed on glass fiber-reinforced PMMA-based matrix composites. Delamination damage was artificially induced during the fabrication of specimens. Thermal healing parameters (temperature and time) were determined experimentally. The delamination area was measured before and after healing by the active thermography technique. The effectiveness of healing was determined mechanically utilizing vibration analysis and a post-impact compression test by comparing the properties of the intact, damaged, and healed specimens.

## 2. Material and Experimental Description

### 2.1. Materials and Specimens

For the preparation of the specimens, Interglas unidirectional 220 gr/m^2^ glass filament fabric (Interglas 92145, aero, FK 144, PW, UD) was used from Porcher Industries (Erbach, Germany). For the composite matrix, MMA-based Orthocryl 617H119 lamination resin was used with 617P37 hardener powder from Ottobock (Duderstadt, Germany). Glass fiber composites have been chosen because of their transparency, which allows for the visual evaluation of the delamination area before and after healing.

Specimens have dimensions of 100 mm × 100 mm with a thickness of 1.5 mm. The lamination process was carried out in two steps. In the first step, glass fiber layers were stacked with a sequence of [90/0/0]. The composite laminate was produced by a wet layup followed by a vacuum bag assisted molding and left to cure overnight at room temperature. In the second step, two fully curried pieces were bonded together to obtain a panel with a laminated sequence of [(90/0/0)/(0/0/90)]. Artificial delamination damage was created by leaving 50 mm diameter unstained areas in the center of the laminates. During bonding, an additional pressure of 0.5 MPa was applied. Due to the higher viscosity of thermoplastic resin compared to epoxy resins, the manufacture of glass fiber-reinforced thermoplastic composite laminates can introduce voids into the composite material, which significantly affects the mechanical properties of the composite laminate. The fiber volume fraction and void content were determined from the fiber-to-resin weight ratio to be 34.7% and 16.0%, respectively. To address any doubts about the experimental results, the Role of Mixture approach was concurrently employed.

Madsen and Lilholt [34] proposed a simple model in which the stiffness reduction caused by voids is represented as a second-order polynomial function of the void volume fraction in both the fiber direction (*E*_1_) and the transverse direction (*E*_2_). The same equations have been used for strength properties by replacing *E* with σ. For transverse strength, the elongation at break of the matrix has been considered:

(1) $$E_{1}=\left(E_{f}V_{f}+{V_{m}E}_{m}\right){\left({1-V}_{v}\right)}^{2}$$

(2) $$E_{2}=\frac{E_{f}E_{m}}{{V_{m}E}_{f}+V_{f}E_{m}}{\left({1-V}_{v}\right)}^{2}$$

where *E_f_*, *E_m_*—Young modulus for fiber and matrix, respectively; *V_f_*, *V_m_*—fiber and matrix volume fraction ratio, respectively; and *V_v_*—voids volume fraction ratio.

The in-plane shear modulus, calculated by the Halpin–Tsai equation [35,36], incorporates the influence of voids

(3) $$G_{12}=\frac{G_{f}G_{m}}{{V_{m}G}_{f}+V_{f}G_{m}}{\left({1-V}_{v}\right)}^{2}$$

where *G_f_*, *G_m_*—the shear modulus for fiber and matrix, respectively.

The ultimate tensile strength in the longitudinal and transverse directions of the unidirectional composite is calculated using a simple model. In the transverse direction, it is assumed to be equal to the matrix tensile strength, accounting for the effect of voids [37].

(4) $$X_{T}=E_{1}\varepsilon_{u}$$

The compressive strength of unidirectional (UD) composites has been assessed using the Budiansky model, which is based on the kinking mechanism [38]. The ratio ∅γy assumed to be equal to 4.

(5) XC=G121+∅γy

(6) $$Y_{T}=\sigma_{mu}\left(1-2\sqrt{\frac{V_{f}}{\pi }}\right)$$

(7) $$DFAILT=\varepsilon_{f}$$

(8) $$DFAILM=\varepsilon_{fu}V_{f}+\varepsilon_{mu}V_{m}$$

The obtained scatter of material constants was extended by 20% and used as a range for material constant calibration. The numerical model of the laminate tensile test has been created in the finite element code LS-Dyna for calibration. Using the LS-OPT 7.0 software, a graphical optimization and parameter identification tool, the material constants have been calibrated, and the values are presented in Table 1. The material constants presented in Table 1 include mechanical properties from data sheets, experiments, micromechanics calculations, the role of mixtures, and calibrated values.

**Figure 1:**
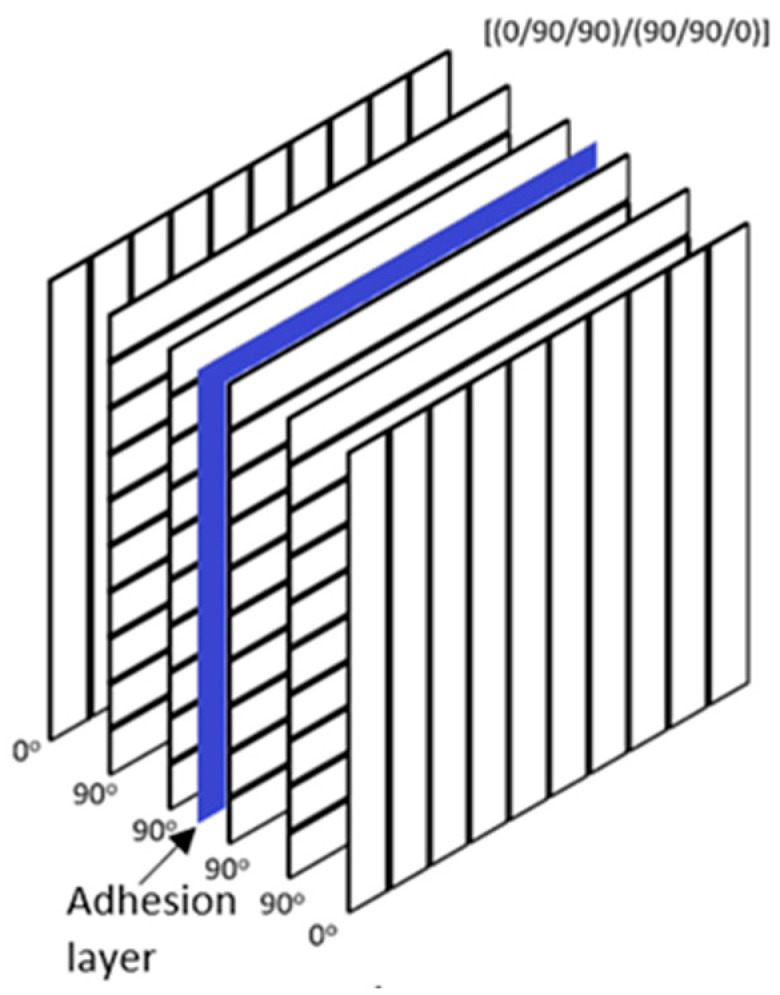
Layup of composite laminate.

Two types of specimens were produced: fully bonded specimens to represent intact structures, and not fully bonded specimens to simulate delamination damage (Figure 1). As described earlier, artificially induced delamination was achieved by intentionally leaving an unbonded space approximately 50 mm in diameter at the center of the plate. This setup resulted in a delamination area of nearly 20%.

### 2.2. Thermal Healing Parameters

Wool and O’Connor [39] developed an experimental approach for crack healing in polymers. The healed material is compared with the virgin material through mechanical or spectroscopic measurements as a function of healing time t_h_ and temperature T_h_. In the current research, these variables were selected to identify the most favorable values of healing parameters that produce the highest delamination recovery effect.

When two sections of the same polymer are joined at a temperature above their glass transition temperature (Tg), the interface gradually disappears, and the mechanical strength at the polymer-polymer interface increases as the crack heals through molecular diffusion [40].

The glass transition temperature of PMMA-based resin was measured via differential scanning calorimetry (DSC). Small sections, cut from molded samples, were measured by Perkin Elmer equipment. DSC showed Tg at 126.8 °C. Accordingly, the delamination recovery procedure was carried out at temperatures no less than 130 °C.

Initially, to determine the temperature effect on the mechanical properties of pure PMMA resin, three-point bending tests were performed on rectangular cross-section specimens with dimensions of 100 × 12 × 18 mm. To evaluate the temperature effect on adhesive interlaminar shear strength, the single lap joints were tested. The specimens for the single-lap joint test were made from glass fiber laminates of 20 mm width bonded together with a 5 mm overlap. All the specimens were cured for 6 different periods of time: 6, 12, 18, 24, 36, and 48 h. It was prepared in 6 specimen groups of 10 specimens. Each group was intended for each curing time.

**Figure 2:**
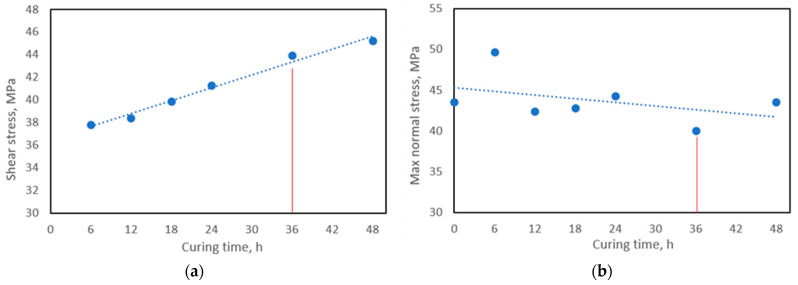
Test results: (**a**) single-lap joint tests result in GFRP shear strength, and (**b**) 3-point bending tests result in pure PMMA resin.

Curing time effect on pure resin strength obtained after three-point bending tests presented in Figure 2b. The curing time did not show any significant effect on the strength properties of pure resin. However, it can be seen that there was no thermal degradation of the resin after curing.

The single-lap joint test (Figure 2a) showed that shear strength increases with curing time. The overall increase was 19.6%, and for further experiments, 36 h of healing time were chosen.

### 2.3. Non-Destructive Testing

**Figure 3:**
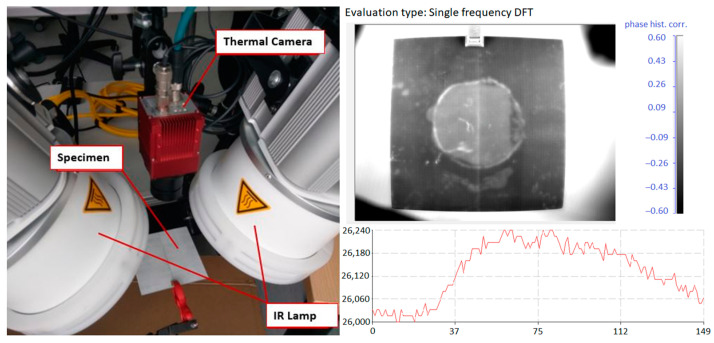
Experimental setup of the lock-in thermography system and IR-NDT v1.7 software interface.

The dimensions of artificially induced delamination and the effect of healing have been determined by applying an active lock-in IR thermography technique. The experimental setup of the lock-in thermography system is presented in Figure 3. Two infrared lamps were used to generate the sine-wave type of excitation heating source signal. The surface temperatures were recorded by a thermal camera and analyzed by IR-NDT v1.7 software.

**Figure 4:**
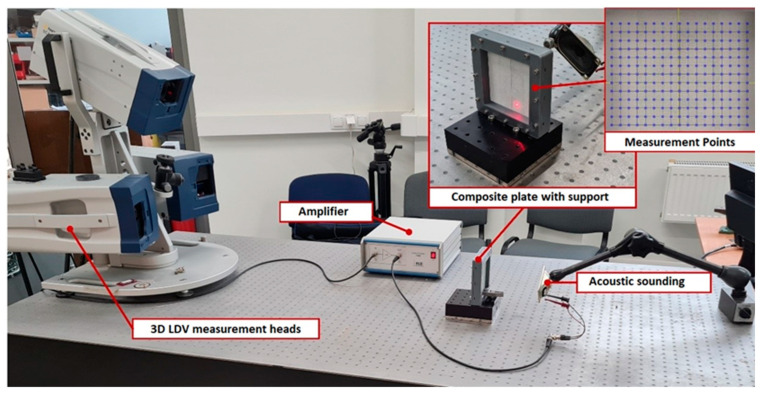
General view of the experimental setup.

Additional NDT methods based on vibration analysis have been utilized to evaluate the healing effect on composite plate properties. The Local Defect Resonance (LDR) approach has been adopted to explore the effect of delamination damage and healing on the dynamical properties of composite laminate. Using the 3D scanning laser Doppler vibrometer, the out-of-plane surface response has been measured to perform the vibration analysis. For plate excitation, piezo-elements are usually used, which are glued to the surface of the samples or on the edges of the samples [32,41,42,43,44], thus a wide frequency range can be excited. Meanwhile, the frequency band of our experimental studies is 100 Hz–10 kHz, so acoustic excitation with a loudspeaker is used, which perfectly generates the required input forces. Additionally, this technique avoids gluing the piezo-elements of the sensors to the surface of the sample. One of the surfaces of the samples is covered with matte paint, because otherwise a high-quality signal reflection is not obtained when measuring with a 3D LDV (Laser Doppler Vibrometer). Equipment used for the experiment is a 3D scanning Laser Doppler vibrometer PSV-W-500 (Polytec GmbH, Karlsbad, Germany), complete with three 3D scanning laser heads (PSV-500). High-voltage and high-current laboratory amplifier P200 (FLC Electronics AB, Gothenburg, Sweden). The general view of the experimental bench is presented in Figure 4, as well as the grid of measurement points. Measurements are performed with chirp excitation, having a frequency band from 100 Hz to 10 kHz, without any filter.

### 2.4. Compression after Impact Test

**Figure 5:**
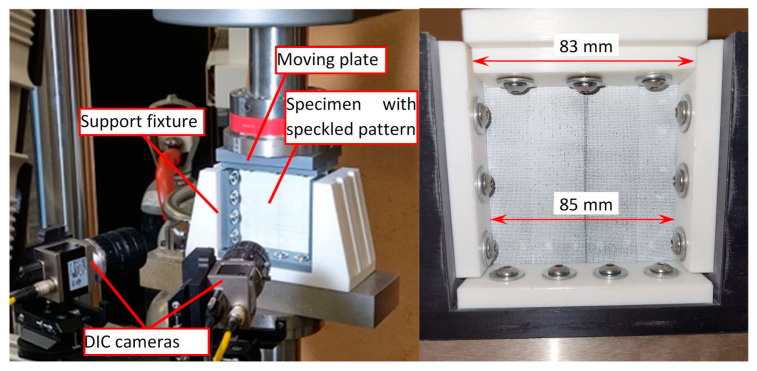
View of CAI testing and 3D-DIC measurement setup.

Interlaminar delamination can be detected through different non-destructive inspection (NDI) techniques. But there is no reliable procedure to quantify the damage recovery, measure the joint strength, or assure that the healed delamination determined by NDT is not the result of “kissing bond” [14]. The compression after impact (CAI) test is adopted to estimate the effect of healing by evaluating the structural response and residual compressive strength of GFRP-laminated plates [45]. The compressive residual strength properties of intact and damaged composite plates were determined according to the ASTM D7137 standard [46]. The tests were carried out on a universal testing machine (ElectroPuls E10000, Instron, Norwood, MA, USA). The specimens were assembled in the standard support fixture (shown in Figure 5) without any antibuckling devices. As the aim of the CAI test was to compare mechanical response, there was no reason to avoid buckling. The assembled support was freely placed on the center of the lower plate. The load was applied using the displacement control method of the top crosshead. The 3D-DIC technique has been applied to analyze the surface deformations and estimate the effect of delamination and healing on the residual strength from the mechanical response of composite plates. The three-dimensional full-field surface displacement and deformation strains were acquired by data acquisition and analyzed by VIC-3D v9.2 software. The displacements of the affected surface of the specimen are mapped simultaneously by two cameras with an optical resolution of 4096 × 3000 pixels, pixel size 3.45 × 3.45 μm, and lenses of focal length 25 mm. The camera’s sampling rate was set at 1 Hz.

There are several definitions to quantify healing efficiency. Wu et al. [47] propose assessing healing using the stiffness recovery ratio, which is obtained by dividing the stiffness of the healed structure by the stiffness of the virgin structure:

(9) $$SRR=\frac{K_{h}}{K_{v}}$$

For comparison, the damage ratio was calculated by dividing the damaged stiffness by the virgin stiffness.

(10) $$DR=\frac{K_{d}}{K_{v}}$$

Blaiszik et al. [25] proposed a more universal equation to evaluate recovery performance (*ƞ*) of any property of interest such as fracture toughness, peak fracture load, strain energy, etc.:

(11) $$\eta =\frac{f_{healed}-f_{damaged}}{f_{virgin}-f_{damaged}}$$

where *f* is any property of the material.

Additionally, finite element analysis using the nonlinear explicit code LS-Dyna was carried out to evaluate the response of the composite plate during the CAI test. A time-scaling approach has been used to simulate quasi-static tests with explicit code. Numerical analysis has been divided into several steps: (a) calibration of the composite material model; (b) calibration of tiebreak contact parameters; (c) validation of CAI numerical models of intact, with delamination damage and healed composite plates.

**Figure 6:**
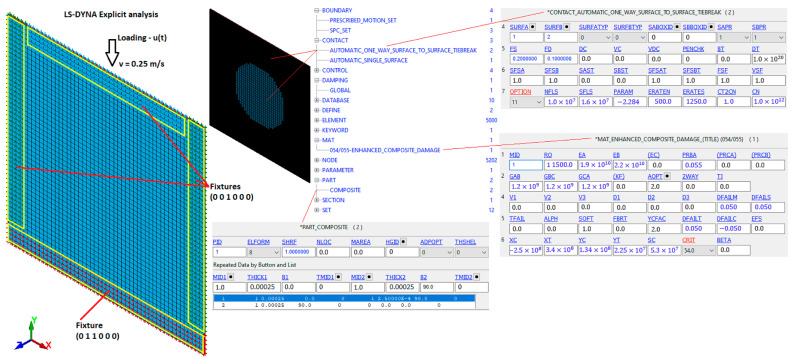
FE model boundary conditions and main input cards of the composite laminate CAI test.

The composite plate was modeled (Figure 6) by two layers of 4-node quadrilateral Belytschko–Leviathan shell elements defined by the *PART_COMPOSITE card. Each shell consists of three UD GFRP plies. The mesh size was 2 mm. The composite material with the option of damage progression was simulated using the material model MAT54 (*MAT_ENHANCED_COMPOSITE_DAMAGE) based on Chang–Chang failure criteria [48] with Hashin’s [49] four damage modes. Adhesion between two separate layers was modeled by tiebreak contact, which is similar to the “Cohesive zone modelling” approach. The tiebreak contact keyword (*CONTACT_AUTOMATIC_ONE_WAY_SURFACE_TO_SURFACE_TIEBREAK) based on stress failure allows for the prediction of mechanical behavior of intact, damaged, and healed composite laminates. Post-failure in TIEBREAK contacts allows the node to interact with the segment as in traditional non-penetration contacts.

Using a multi-objective optimization procedure embedded in LS-OPT 7.0 software, the parameter identification approach was adopted to determine the tiebreak contact parameters. The force vs. displacement curves and out-of-plane displacement have been used as criteria to validate the finite element models of CAI tests.

## 3. Results and Discussion

**Figure 7:**
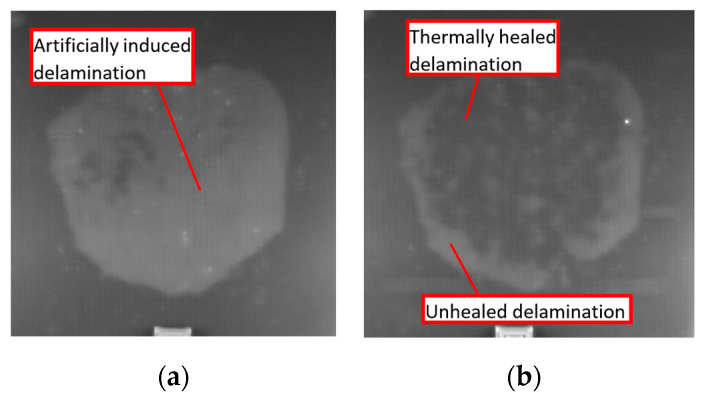
FE Experimental results of lock-in IR thermography (**a**) composite plate before healing; (**b**) after healing model boundary conditions and main input cards of composite laminate CAI test.

From the thermography analysis, the presence of a healing effect is seen (Figure 7). Indeed, the results do not allow us to quantitatively evaluate the strength of the adhesion joint and confirm that healed areas are not a consequence of the kissing bond. It is also seen that not all the delamination damage has been completely healed. Thin glass fiber-reinforced composite samples being transparent allows us to confirm that active thermography is able to precisely determine the healed zone and remaining delaminated regions. Based on the results obtained by active thermography, the FE models of healed samples can be developed.

**Figure 8:**
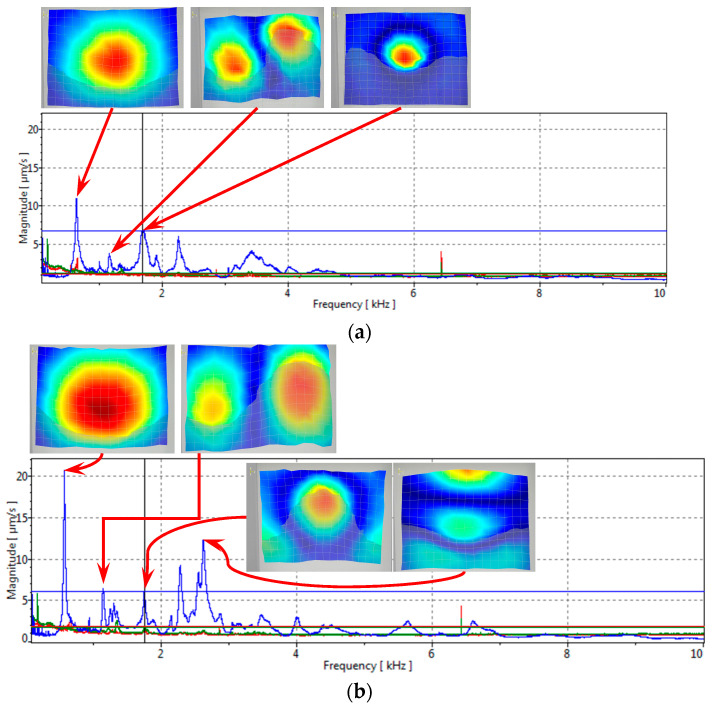
Results of modal experimental analysis (blue lines are velocities in out of plane direction, red and green in plane velocities); (**a**) composite laminate with delamination damage and (**b**) laminate vibration after thermal healing. Modes shape colors are auto scaled for each peak.

The results of vibration analysis (Figure 8) show the differences in amplitudes of surface velocities. It can be seen that the 1st resonant mode is the most pronounced, but in the healed sample, the amplitude of the first resonant mode is about 20–25% higher than in the samples with artificially simulated delamination damage. It confirms that healed specimens, being stiffer in the out-of-plane direction, exhibit higher surface velocities. The observed frequencies between the two samples do not exhibit a significant change. The minimum resolution of the setup allows for the capture of frequencies up to 100 Hz, resulting in no observable changes in frequency values, i.e., the change in frequencies is less than 100 Hz. Nevertheless, a notable alteration in value may be detected when comparing the third peak of the spectrum of the delaminated sample to that of the healed sample. Figure 8a illustrates the presence of a third peak at approximately 1600 Hz, while the healed sample exhibited a peak in the vicinity of 1700 Hz. This finding confirms the increase in stiffness value after the healing process. In the higher frequency range, specifically in the fourth and fifth modes, a significant vibration mode with natural frequencies at 2.7 kHz appears in the cured sample but is absent in the sample with delamination. This suggests the need to analyze higher-order natural frequency oscillation characteristics to assess the healing of the delamination defect.

The applied NDT methods allow for the evaluation of the regions of the healed zone and indicate if the stiffness of the damaged zone has increased. To evaluate the effectiveness of the damage recovery process, a mechanical test is needed.

**Figure 9:**
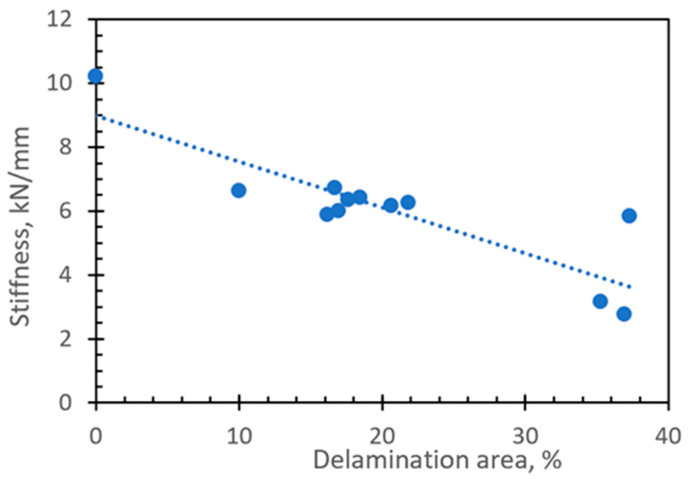
CAI test results presenting the effect of delamination area size on longitudinal stiffness.

The specimens with artificially induced delamination damage were formed manually, which resulted in different sizes of delamination areas. Comparing the force vs. displacement curves (Figure 9) obtained from the CAI test, it was revealed that there is a correlation between the longitudinal stiffness of the composite plate and the delamination area. The stiffness of the specimen decreases with the increase in the size of the delamination area.

**Figure 10:**
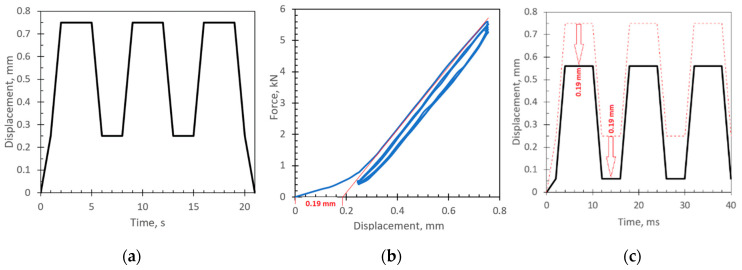
CAI test (**a**) experimental loading path controlled by displacement (**b**) experimentally obtained force vs. displacement curve (**c**) loading patch for FE model (black curve) with subtracted initial nonlinearity (0.19 mm) obtained during the experimental (dashed curve) testing.

The CAI test up to failure was conducted on three distinct batches of intact, damaged, and healed samples. The obtained maximum forces of 9.5 ± 0.28 kN, 7.4 ± 0.37 kN, and 8.0 ± 0.32 kN and displacements at maximum forces of 1.09 mm, 1.15 mm, and 1.1 mm, respectively were used to define the loading parameters for further tests. To avoid the effect of delamination parameters like size and shape, nondestructive loading was chosen based on the CAI test. The loading amplitudes were chosen to be up to 75% of the limit displacement values. This allows each sample to be tested twice to compare behavior with delamination damage and after healing. Firstly, the samples with artificial damage were tested; later, after the healing procedure for the same sample, the CAI test was repeated. All samples were tested using the same loading patch pattern (Figure 10a), which consists of three cycles controlled by the displacement channel. The loading of more than two cycles was chosen just to ensure that the destruction of healing did not appear during the first loading step. The obtained experimental force vs. displacement results show nonlinearity at the beginning of the first cycle (Figure 10b), which could be affected by geometrical imperfections of the specimens and bearing deformations at the contact regions. As during the cycling loading the samples stay stressed all the time, the initial nonlinearity, which in this particular case was equal to 0.19 mm, has been subtracted from the loading patch used for FE simulation (Figure 10c). Figure 10b shows the effect of healing and stiffness degradation on the first steps of cycling loading.

**Figure 11:**
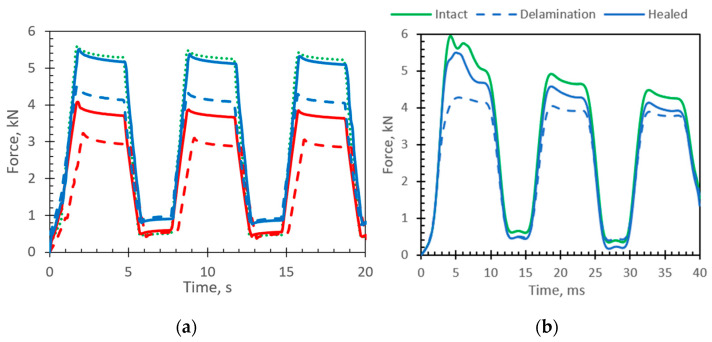
CAI test results: (**a**) experimental curves (dot green curve represents an intact specimen; dashed curves represents specimens with delamination; solid curves represent healed specimens (red color—37% of delamination area, blue color—10% of delamination area); (**b**) FE simulation (green curve represents an intact specimen).

Typical responses of intact, damaged, and healed specimens to the cycling loading patch (Figure 10a) are presented in Figure 11. The results (Figure 11a) show the effect of healing and stiffness degradation on the first steps of cycling loading. Across all specimens, the average stiffness healing effectiveness, which was evaluated by Equation (11), was 46%. Through all specimens, the average stiffness recovery ratio (Equation (9)) was 73%, while the average damage ratio obtained by Equation (10) was 55%.

The diminished response of the healed samples is a consequence of either damage accumulation in the cohesion zone of the healed area or the incomplete healing of the entire delaminated region. During the experiments, the level of loading was selected to avoid inducing damage to the specimens. However, it is likely that the weaker response of the healed specimen is a result of incomplete healing of the delamination (see Figure 7b). In this context, for the numerical modeling, the healed delamination area has been reduced, leaving a gap of approximately 5 mm. In numerical modeling, the properties of the cohesion zone are simulated by tiebreak contact, which is determined by three parameters: failure stresses (normal NFLS and shear SFLS), energy release rates (normal ERATEN and shear ERTATES), and contact stiffnesses (normal and tangential). A high scatter of PMMA energy release rate values can be found in the literature [50,51,52]. Mostly, the ratio between the shear and normal components can be found in the range between 2.3 and 2.8. To decrease the number of variables, it was assumed that the ratio between shear and normal energy release rate is equal to 2.5, and failure stresses have been obtained from the numerical stability condition. It was assumed that the number of finite elements in the cohesive zone would not be less than 3 [53]. Contact stiffnesses were assumed to be equal and calibrated according to the experimental results.

**Figure 12:**
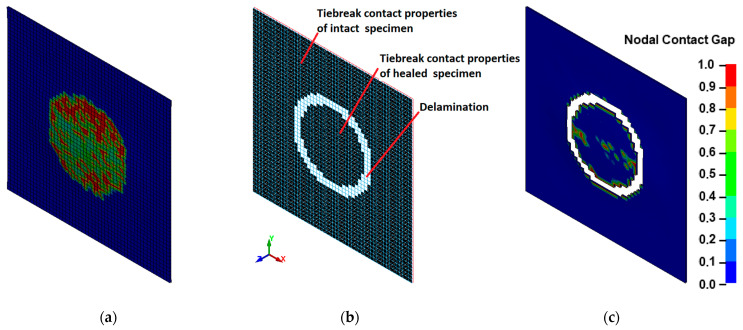
FE modeling of healed specimens: (**a**) delamination damage distribution in the healed region; (**b**) two tiebreak contact segments having the properties of intact and healed samples; (**c**) delamination damage distribution in the case of an incompletely healed specimen (like in Figure 7b).

The delamination damage distribution seen in Figure 12a does not match the experimental results. After the CAI test of the healed specimens, the growth of delamination damage was not observed. The results of the FE model with an incompletely healed specimen corelate from experiments indicate that small areas of delamination damage start in the unhealed region. The results of the numerical analysis show that a calibrated FE model allows for the evaluation of the delamination effect on the stress state and the prediction of the delamination growth of damaged and healed laminate. After the identification and calibration of material and adhesion parameters, the numerical models can be adopted for application in practical scenarios to evaluate the structural integrity of damaged and healed composite structures.

Experimental results show that the size of the delamination remained almost unchanged after the healed samples were subjected to multiple load cycles. Further, the partially restored stability and strength indicate that bonds formed through interdiffusion are strong. It confirms that for continuous fiber-reinforced PMMA-based composites and the interdiffusion theory [54,55], two polymer surfaces being in contact with each other will interdiffuse if the polymer chains are heated above the glass transition temperature and become sufficiently mobile to conduct self-bonding.

**Figure 13:**
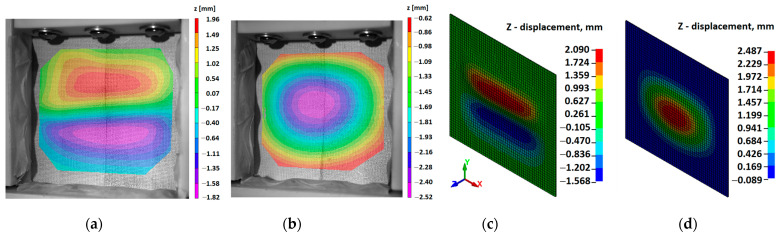
Out-of-plane displacement obtained during the CAI test: (**a**) DIC results of specimens with delamination damage; (**b**) DIC results of intact specimens; (**c**) FE results of specimens with delamination damage; and (**d**) FE results of intact specimens.

A comparison is shown between CAI, DIC, and simulation results in Figure 13. Here, one can observe that the surface displacement characteristic of a delaminated and intact sample perfectly matches the simulation results. The maximum value of surface displacement is estimated at 1.96 mm from DIC, whereas the simulation results show a maximum displacement of 2.09 mm for the delaminated sample. Similarly, the minimum value was estimated at 1.82 mm and 1.57 mm from DIC and simulation, respectively (shown in Figure 13a,c). On the other hand, the absolute maximum surface displacement for an intact sample is estimated at 2.52 mm by DIC, and simulation results give the same parameter as 2.49 mm. These values confirm the correctness of the finite element model.

## 4. Conclusions

The recovery of the delamination in glass fiber-reinforced thermoplastic composites has been studied experimentally and numerically under loading according to the compression-after-impact methodology. The same process is validated with two NDT methods. It was shown that delamination damage artificially induced during the fabrication of laminate plates can be healed by thermal heating, applying the temperature slightly above the glass transition point.

- The non-destructive testing by active thermography was useful to determine the size of delamination and see the presence of thermal healing, but it did not allow for a quantitative evaluation of the strength of the adhesion joint and confirm that healed areas are not the consequence of the kissing bond. Dynamical analysis performed by the 3D Laser Doppler Vibrometer shows the differences in amplitudes of surface velocities. The healed specimens having about 20% higher amplitudes compared to specimens with delamination confirms that the healed specimens being stiffer in the out-of-plane direction exhibit higher surface velocities;
- Comparing experimentally obtained force vs. displacement curves, the correlation between the longitudinal stiffness of the composite plate and the delamination area was obtained. The stiffness of the specimen decreases with the increase in the size of the delamination area. Comparing the experimentally obtained responses of damaged and healed specimens to the cycling loading, the average effectiveness of healing was 29%;
- To perform numerical analysis of composite plate delamination, the finite element model was developed with two layers of shell elements connected by tiebreak contact. The numerical model effectively captured the experimentally obtained shapes of the first buckling mode, which differs for intact and damaged specimens. The variation of reaction force well matches the experimentally obtained response. In cases of incompletely healed regions, the delamination damage starts around the unhealed areas and propagates towards the most stressed regions. It can be concluded that a numerical model with a tiebreak contact approach could be used to simulate and predict the behavior of intact, damaged, and healed composite plates.

Analysis of the entire surface strain field captured by the 3D DIC technique indicates the possibility of detecting internal damage. A more comprehensive analysis of healing effectiveness on damage types, size, depth, and fatigue life looks relevant for future research.

## Figures and Tables

**Table 1:** Mechanical properties of materials.

Parameter		Units	Glass Fiber	PMMA (Exp)	UD Composite Ply		
					Exp	ROMMicromechanics	Calibrated
Density	ρ	g/cm^3^	2.60 *	1.185	1.47		
Fiber volume fraction	$V_{f}$				0.34		
Matrix volume fraction	$V_{m}$				0.50		
Void volume fraction	$V_{v}$				0.16		
Tensile modulus	$E_{1}$	GPa	73 *	2.45	23.2	18.4	19.0
	$E_{2}$				2.1	2.53	2.2
Poisson ratio	$v_{12}$		0.18 *	0.4	0.18	0.26	0.26
Shear modulus	$G_{12}$	GPa	30.9	0.875	-	1.21	1.18
Tensile strength	$X_{T}$	MPa	3400 *	45	325	643	340
	$Y_{T}$				16.7	15.4	22.5
Compressive strength	$X_{C}$	MPa		117		242	250
	$Y_{C}$						134
Shear strength	$S_{12}$	MPa					53
Elongation at break	*DFAILT*	%	3.5 *	1.9		3.5	2.6
	*DFAILC*	%					2.5
	*DFAILM*	%				2.1	4.0

* parameters are taken from manufacturers datasheets.

## Data Availability

The data presented in this study are available upon request from the corresponding authors. The data are not publicly available due to privacy.

## References

[1] Cousins D.S., Suzuki Y., Murray R.E., Samaniuk J.R., Stebner A.P. (2019). Recycling glass fiber thermoplastic composites from wind turbine blades. J. Clean Prod..

[2] Stewart R. (2011). Thermoplastic composites—Recyclable and fast to process. Reinf. Plast..

[3] Yousefpour A., Hojjati M., Immarigeon J.-P. (2004). Fusion Bonding/Welding of Thermoplastic Composites. J. Thermoplast. Compos. Mater..

[4] (2013). Process and Performance Evaluation of Ultrasonic, Induction and Resistance Welding of Advanced Thermoplastic Composites—Irene Fernandez Villegas, Lars Moser, Ali Yousefpour, Peter Mitschang, Harald EN Bersee. https://journals.sagepub.com/doi/10.1177/0892705712456031.

[5] Robles J.B., Dubé M., Hubert P., Yousefpour A., Dub M. (2022). Repair of thermoplastic composites: An overview. Adv. Manuf. Polym. Compos. Sci..

[6] Nijhuis P. (2013). Repair of thin walled thermoplastic structures by melting, an experimental research. Seico.

[7] Khan T., Hafeez F., Umer R. (2023). Repair of Aerospace Composite Structures Using Liquid Thermoplastic Resin. Polymers.

[8] Bhudolia S.K., Perrotey P., Joshi S.C. (2018). Mode I fracture toughness and fractographic investigation of carbon fibre composites with liquid Methylmethacrylate thermoplastic matrix. Compos. B Eng..

[9] Pini T., Caimmi F., Briatico-Vangosa F., Frassine R., Rink M. (2017). Fracture initiation and propagation in unidirectional CF composites based on thermoplastic acrylic resins. Eng. Fract. Mech..

[10] Wong J., Altassan A., Rosen D.W. (2003). Additive manufacturing of fiber-reinforced polymer composites: A technical review and status of design methodologies. Compos. B Eng..

[11] Obande W., Brádaigh C.M.Ó., Ray D. (2021). Continuous Fibre-Reinforced Thermoplastic Acrylic-Matrix Composites Prepared by Liquid Resin Infusion—A Review. Compos. B Eng..

[12] Khan T., Irfan M.S., Cantwell W.J., Umer R. (2022). Crack healing in infusible thermoplastic composite laminates. Compos. Part A Appl. Sci. Manuf..

[13] Ouyang T., Sun W., Bao R., Tan R. (2021). Effects of matrix cracks on delamination of composite laminates subjected to low-velocity impact. Compos. Struct..

[14] Li W., Palardy G. (2022). Damage monitoring methods for fiber-reinforced polymer joints: A review. Compos. Struct..

[15] Sam-Daliri O., Faller L.M., Farahani M., Zangl H. (2021). Structural health monitoring of adhesive joints under pure mode I loading using the electrical impedance measurement. Eng. Fract. Mech..

[16] Sam-Daliri O., Faller L.-M., Farahani M., Roshanghias A., Araee A., Baniassadi M., Oberlercher H., Zangl H. (2019). Impedance analysis for condition monitoring of single lap CNT-epoxy adhesive joint. Int. J. Adhes. Adhes..

[17] Frederick H., Li W., Sands W., Tsai E., Palardy G. Multifunctional Films For Fusion Bonding And Structural Health Monitoring of Thermoplastic Composite Joints. Proceedings of the SAMPE 2020 Virtual Series.

[18] Hua J., Xing S., An S., Chen D., Tang J. (2022). Stitching Repair for Delaminated Carbon Fiber/Bismaleimide Composite Laminates. Polymers.

[19] Cang Y., Hu W., Zhu D., Yang L., Hu C., Yuan Y., Wang F., Yang B. (2022). In Situ Thermal Ablation Repair of Delamination in Carbon Fiber-Reinforced Thermosetting Composites. Energies.

[20] Ageorges C., Ye L., Hou M. (2021). Advances in fusion bonding techniques for joining thermoplastic matrix composites: A review. Compos. Part A Appl. Sci. Manuf..

[21] Aïssa B., Therriault D., Haddad E., Jamroz W. (2012). Self-Healing Materials Systems: Overview of Major Approaches and Recent Developed Technologies. Adv. Mater. Sci. Eng..

[22] Post W., Cohades A., Michaud V., van der Zwaag S., Garcia S.J. (2017). Healing of a glass fibre reinforced composite with a disulphide containing organic-inorganic epoxy matrix. Compos. Sci. Technol..

[23] Javierre E. (2019). Modeling self-healing mechanisms in coatings: Approaches and perspectives. Coatings.

[24] Wang S., Urban M.W. (2020). Self-healing polymers. Nat. Rev. Mater..

[25] Blaiszik B.J., Kramer S.L.B., Olugebefola S.C., Moore J.S., Sottos N.R., White S.R. (2010). Self-healing polymers and composites. Annu. Rev. Mater. Res..

[26] Pittala R.K., Dhanaraju G., Ben B.S., Ben B.A. (2022). Self-healing of matrix cracking and delamination damage assessment in microcapsules reinforced carbon fibre epoxy composite under flexural loading. Compos. Struct..

[27] Ahangaran F., Hayaty M., Navarchian A.H., Pei Y., Picchioni F. (2019). Development of self-healing epoxy composites via incorporation of microencapsulated epoxy and mercaptan in poly(methyl methacrylate) shell. Polym. Test.

[28] Patrick J.F., Hart K.R., Krull B.P., Diesendruck C.E., Moore J.S., White S.R., Sottos N.R. (2014). Continuous self-healing life cycle in vascularized structural composites. Adv. Mater..

[29] Norris C.J., Bond I.P., Trask R.S. (2011). The role of embedded bioinspired vasculature on damage formation in self-healing carbon fibre reinforced composites. Compos. Part A Appl. Sci. Manuf..

[30] Pittala R.K., Ben B.S., Ben B.A. (2021). Self-healing performance assessment of epoxy resin and amine hardener encapsulated polymethyl methacrylate microcapsules reinforced epoxy composite. J. Appl. Polym. Sci..

[31] Chuang Y.F., Wu H.C., Yang F., Yang T.J., Lee S. (2016). Cracking and healing in poly(methyl methacrylate): Effect of solvent. J. Polym. Res..

[32] Post W., Kersemans M., Solodov I., Van Den Abeele K., García S.J., van der Zwaag S. (2017). Non-destructive monitoring of delamination healing of a CFRP composite with a thermoplastic ionomer interlayer. Compos. Part A Appl. Sci. Manuf..

[33] Kanu N.J., Gupta E., Vates U.K., Singh G.K. (2019). Self-healing composites: A state-of-the-art review. Compos. Part A Appl. Sci. Manuf..

[34] Madsen B., Lilholt H. (2003). Physical and mechanical properties of unidirectional plant fibre composites—An evaluation of the influence of porosity. Compos. Sci. Technol..

[35] Shokrieh M.M., Moshrefzadeh-Sani H. (2016). On the constant parameters of Halpin-Tsai equation. Polymer.

[36] Affdl J.C.H., Kardos J.L. (1976). The Halpin-Tsai equations: A review. Polym. Eng. Sci..

[37] Naik N.K., Kumar R.S. (1999). Compressive strength of unidirectional composites: Evaluation and comparison of prediction models. Compos. Struct..

[38] Budiansky B. (1983). Micromechanics. Comput. Struct..

[39] Wool R.P., O’Connor K.M. (1981). A theory crack healing in polymers. J. Appl. Phys..

[40] Lin C.B., Lee S., Liu K.S. (1990). Methanol-Induced crack healing in poly(methyl methacrylate). Polym. Eng. Sci..

[41] Wei L., Chen J. (2022). Characterization of delamination features of orthotropic CFRP laminates using higher harmonic generation technique: Experimental and numerical studies. Compos. Struct..

[42] Segers J., Kersemans M., Hedayatrasa S., Calderon J., Van Paepegem W. (2018). Towards in-plane local defect resonance for non-destructive testing of polymers and composites. NDT E Int..

[43] Solodov I., Kreutzbruck M. (2020). Ultrasonic frequency mixing via local defect resonance for defect imaging in composites. Ultrasonics.

[44] Segers J., Kersemans M., Verboven E., Hedayatrasa S., Calderon J., Van Paepegem W. (2018). Investigation to local defect resonance for non-destructive testing of composites. Proceedings.

[45] Lu T., Shen H.-S., Wang H., Chen X. (2022). Compression-after-impact effect on postbuckling behavior of thermoplastic composite laminated plates. Aerosp. Sci. Technol..

[46] (2007). Standard Test Method for Compressive Residual Strength Properties of Damaged Polymer Matrix Composite Plates.

[47] Wu X.-F., Rahman A., Zhou Z., Pelot D.D., Sinha-Ray S., Chen B., Payne S., Yarin A.L. (2012). Electrospinning core-shell nanofibers for interfacial toughening and self-healing of carbon-fiber/epoxy composites. J. Appl. Polym. Sci..

[48] Chang F.-K., Chang K.-Y. (1987). A Progressive Damage Model for Laminated Composites Containing Stress Concentrations. J. Compos. Mater..

[49] Hashin Z. (1980). Failure Criteria for Unidirectional Fiber Composites. J. Appl. Mech..

[50] Tabiei A., Zhang W. A Zero Thickness Cohesive Element Approach for Dynamic Crack Propagation using LS-DYNA^®^. Proceedings of the 15th International LS-DYNA Conference.

[51] Dempsey J.P., Wei Y. (1989). Fracture Toughness of S2 Columnar Freshwater Ice: Crack Length and Specimen Size Effects-Part II. https://www.researchgate.net/profile/John-Dempsey-4/publication/304010858_Fracture_toughness_of_S2_columnar_freshwater_ice_crack_length_and_specimen_size_effects_-_Part_II/links/5762a65008aee61395bef19a/Fracture-toughness-of-S2-columnar-freshwater-ice-crack-length-and-specimen-size-effects-Part-II.pdf.

[52] Yu R.C., Pandolfi A., Ortiz M. (2007). A 3D cohesive investigation on branching for brittle materials. IUTAM Symposium on Discretization Methods for Evolving Discontinuities.

[53] Turon A., Davila C.G., Camanho P.P., Costa J. (2007). An engineering solution for mesh size effects in the simulation of delamination using cohesive zone models. Eng. Fract. Mech..

[54] Aradian A., Raphaël E., De Gennes P.G. (2000). Strengthening of a polymer interface: Interdiffusion and cross-linking. Macromolecules.

[55] Awaja F. (2016). Autohesion of polymers. Polymer.

